# The effects of a motorized passive simulated jogging device on descent of the arterial pulse waveform dicrotic notch: A single arm placebo‐controlled cross‐over trial

**DOI:** 10.14814/phy2.15418

**Published:** 2022-08-03

**Authors:** Jose A. Adams, Jose R. Lopez, Vinay Nadkarni, Zarazuela Zolkipli‐Cunningham, Harry Ischiropoulos, Marvin A. Sackner

**Affiliations:** ^1^ Division Neonatology Mt Sinai Medical Center of Greater Miami Miami Beach Florida USA; ^2^ Department of Research Mt Sinai Medical Center of Greater Miami Miami Beach Florida USA; ^3^ Anesthesiology, Critical Care, and Pediatrics, The Children's Hospital of Philadelphia University of Pennsylvania Perelman School of Medicine Philadelphia Pennsylvania USA; ^4^ Mitochondrial Medicine Frontier Program (MMFP), Center for Mitochondrial and Epigenomic Medicine (CMEM), Division of Human Genetics, The Children's Hospital of Philadelphia University of Pennsylvania Perelman School of Medicine Philadelphia Pennsylvania USA; ^5^ Children's Hospital of Philadelphia Research Institute and Division of Neonatology, Departments of Pediatrics and Systems Pharmacology and Translational Therapeutics, the Raymond and Ruth Perelman School of Medicine University of Pennsylvania Philadelphia Pennsylvania USA

**Keywords:** dicrotic notch, gentle jogger, nitric oxide, passive simulated jogging, physical activity, pulsatile shear stress, pulse waveform analysis

## Abstract

Whole Body Periodic Acceleration (WBPA, pGz), is a bed that moves the body headward to forward, adds pulses to the circulation inducing descent of the dicrotic notch (DN) on the pulse waveform with an increase in a/b ratio (a = the height of the pulse waveform and b = the height of the secondary wave). Since the WBPA is large, heavy, and non‐portable, we engineered a portable device (Jogging Device, JD). JD simulates passive jogging and introduces pulsations to the circulation. We hypothesized that JD would increase the a/b ratio during and after its use. In Study A, a single‐arm placebo‐controlled cross‐over trial was conducted in24 adults (53.8 ± 14.4 years) using JD or control (CONT) for 30 min. Blood pressure (BPs and BPd) and photoplethysmograph pulse (a/b) were measured at baseline (BL), during 30 min of JD or CONT, and 5 and 60 min after. In Study B (*n* = 20, 52.2 ± 7 years), a single‐arm observational trial of 7 consecutive days of JD on BP and a/b, measured at BL, and after 7 days of JD and 48 and 72 hr after its discontinuation. In Study A, BPs, and BPd decreased during JD by 13% and 16%, respectively, while in CONT both increased by 2% and 2.5%, respectively. The a/b increased by 2‐fold and remained greater than 2‐fold at all‐time points, with no change in a/b during CONT. In Study B, BPs and BPd decreased by 9% and remained below BL, at 72 hr after discontinuation of JD. DN descent also occurred after 7 days of JD with a/b increase of 80% and remained elevated by 60% for at least 72 h. JD improves acute and longer‐term vascular hemodynamics with an increase in a/b, consistent with increased effects of nitric oxide (NO). JD may have significant clinical and public health implications.

## Graphical abstract

A simple effortless passive simulated jogging device (JD), that induces pulsatile shear stress, with a resultant decrease in dicrotic notch position on the pulsewaveform (consistent with an increase in nitric oxide bioavailability)was studied in adult participants. JD both acutely and used for 7 consecutive days decreases both systolic and diastolic blood pressure and increases nitric oxide bioavailability.

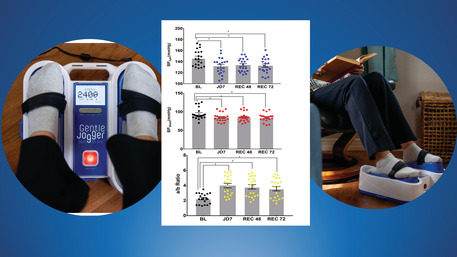

## 1 INTRODUCTION

One of the most important benefits of exercise for health and disease is the increase in nitric oxide (NO) bioavailability (Adams & Linke, 2019; Green et al., 2004; Nosarev et al., 2014; Schuler et al., 2013). Exercise increases NO bioavailability through pulsatile shear stress‐induced phosphorylation (activation) of endothelial nitric oxide synthase (eNOS) (Balligand et al., 2009; Green et al., 2004; Hambrecht et al., 2003; Park et al., 2019; Tran et al., 2022; Zhang et al., 2009). NO generated from eNOS has vasodilator, anti‐atherosclerotic, anti‐inflammatory, angiogenic and metabolic actions (Bahadoran et al., 2020; Farah et al., 2018; Tenopoulou & Doulias, 2020). We have previously shown that a passive exercise device, Whole Body Periodic Acceleration (WBPA), performed with a motion platform, which moves the supine body headward to footward (frequency of 120–140 cycles per minute and acceleration of ±0.5 m/s^2^ in the Z plane) induces pulsatile shear stress in the endothelium, increasing NO (Rokutanda et al., 2011; Sackner et al., 2005a; Sackner et al., 2005b; Uryash et al., 2009; Wu et al., 2012). We have also validated a pulse waveform analysis method to correlate the descent of the dicrotic notch (DN) with vasodilatation related to an increase in the expression of NO and eNOS in anesthetized rats (Uryash et al., 2009). The WBPA is large (size of a single bed), heavy and non‐portable, therefore, a portable predicate device (Gentle Jogger, a.k.a. The Jogging Device (JD), Sackner Wellness Products LLC, Miami Fl, USA) which weighs less than 20 lbs (10 kg), was engineered to deliver pulsatile shear stress in seated and supine postures. The JD is a low‐risk wellness device that meets the requirements of the Food and Drug Administration (FDA) as such [General Wellness: Policy for Low‐Risk Devices Guidance for Industry and Food and Drug Administration Staff, September 27, 2019]. We tested the ability of JD to increase the descent of the dicrotic notch using our previously validated pulse waveform analysis method.

The dicrotic notch or wave of the digital pulse down the diastolic limb reflects the vasodilator actions of NO on resistance vessels due to delay in the reflection of the pulse wave. The latter has been shown with endothelial‐independent preparations of organic nitrates, as well as with endothelial‐dependent agents such as albuterol and terbutaline, ß–adrenergic agents that work via the NO pathway (Klemsdal et al., 1994; Nier et al., 2001; Nier et al., 2008; Weinberg, Habens, Kengatharan, Barnes, & Matz, 2001; Weinberg, Habens, Kengatharan, Barnes, Matz, Anggard, & Carrier, 2001). We have previously shown that 10 min of JD acutely increases NO (Adams et al., 2021). In this study, we hypothesized that JD would acutely increase the descent of the dicrotic notch in a time‐dependent manner during and after its use.

## 2 METHODS

### 2.1 IRB approval for simulated passive jogging device & NO

These investigations and their informed consents were approved by the Western Institutional Review Board (WIRB), Study Numbers: 11172318, GJHP03122018 and WIRBs: 20170208374, 20180772 (WIRB, Puyallup, WA 98374–2115). Trials were registered on ClinicalTrials.gov (NCT03426774 and NCT03550105). These studies are a subunit of two larger protocols in which multiple postures and glycemic effects of JD were investigated. There were two parts in the present study; Study A) Acute effects of JD on blood pressure and position of DN, Study B) Effects of 7 day use of JD (at least three times per day for 30 min for 7 days) on blood pressure and position of DN. Study A was designed as a single‐arm placebo controlled cross‐over trial, in seated posture, with each subject serving as his or her own control (Figure S1 and S2). Study B was also a subunit of a larger observational study in which 20 subjects participated in a longer‐term use of JD and the effects of JD were measured on blood pressure and DN. (Figure S3 and S4) The Consolidated Standards of Reporting Trials (CONSORT) Checklist is found in Supplemental data file (Supplemental data).

### 2.2 Participants

Study A (Acute Effects of JD on Blood Pressure and DN position); Twenty‐four ambulatory individuals consisting of 15 women and 9 men were recruited for this study by word of mouth and gave their informed consent to participate. Their mean age was 53.7 SD 14.4 years and mean BMI of 28.0, SD 4.3. They fasted for at least 8 h and were asked not to drink coffee the day before their participation. The BMI was computed to characterize participants as follows: BMI normal weight 18.5–24.9, overweight 25–29.9 and obese 30 or more. Four women had normal BMI, four were overweight, and seven were obese. For men, two had a normal BMI, five were overweight, and two were obese. The studies were carried out in the morning (Table 1). Study B (Effects of 7 day use of JD on blood pressure and DN position); This study recruited a separate cohort of volunteers (*n* = 20) with characteristics similar to those of Study A. Their mean age was 52.2, SD 17 years and mean BMI 27.2, SD 4. Four women had a normal BMI, five were overweight and three were obese. For men, one had a normal BMI, five were overweight, and two were obese. These studies were also carried out in the morning (Table 2).

**TABLE 1:** Participant's characteristics study A; acute effects of JD on blood pressure and DN position

Subject No	Gender	Age Range (years)	BMI (Kg/mt^2^)	BMI Status	HTN Status	Medications
1	M	50–55	27.8	OV	G 2–3	Metoprolol/Metformin/Losartan
2	F	45–50	30.3	OB	HNL	Lisinopril/Metformin/Levothyroxine
3	F	47–52	31.4	OB	HNL	
4	F	36–41	32.3	OB	NL	
5	F	47–52	29.1	OV	NL	
6	M	26–31	29.8	OV	G 2–3	
7	F	40–45	20.9	NL	NL	
8	F	26–31	20.5	NL	NL	
9	F	33–38	23.2	NL	NL	
10	F	39–44	33.5	OB	G 2–3	
11	F	42–47	35.4	OB	G 2–3	
12	M	23–28	19	NL	NL	
13	F	42–47	30	OB	NL	
14	M	55–60	29.3	OV	G 2–3	
15	M	63–68	30.7	OB	HNL	
16	F	85–88	31.1	OB	G 1	Atenolol/Amlodipine
17	M	56–61	24	NL	G 2–3	Metoprolol
18	F	56–61	29.6	OV	HNL	Lisinopril/Insulin
19	F	59–64	27.2	OV	G 1	Lisinopril/Metformin
20	F	63–68	23.2	NL	G 2–3	Atenolol/Amlodipine
21	F	64–69	28.9	OV	G 2–3	
22	M	63–68	32.1	OB	G2‐3	Atenolol
23	M	58–63	25.8	OV	G 2–3	Atenolol
24	M	55–60	28.2	OV	NL	
Mean ± SD		53.8 ± 14.4	28 ± 4.3	6 = NL, 9 = OV, 9 = OB	8 = N, 4 = HNL, 1 = G1, 11 = G2‐3	

*Notes*: Participant's Characteristics of Study A; Gender (M = male, F = Female), Age Range in years, body mass index (BMI), BMI Status; BMI normal weight BMI 18.5–24.9 (NL), overweight 25–22.9 (OV) and obese 30 or more (OB). The initial blood pressure status (HTN) at the beginning of the study was classified according to the 2021 European guidelines (Stergiou et al., 2021) Normal optimal BP (NL <130/85 mmHg), High‐normal BP (HNL, 130–139/85–89 mmHg), Hypertension grade 1 (G1, 140–159/90–99 mmHg), Hypertension grade 2 and 3 (G2‐3, ≥160/100 mmHg) and medications used at the time of enrollment

**TABLE 2:** Participant's characteristics study B; effects of 7 day use of JD on blood pressure and DN position

Subject No	Gender	Age Range (years)	BMI (Kg/mt^2^)	BMI Status	HTN Status	Medications
1	F	35–45	32.3	OB	NL	
2	F	35–45	20.9	NL	NL	
3	M	60–70	30.7	OB	HNL	
4	F	75–85	31.1	OB	G 1	Atenolol/Amlodipine
5	M	60–70	32.1	OB	G 2	Atenolol
6	F	60–70	29.6	OV	HNL	Lisinopril/Insulin
7	M	50–60	29.3	OV	G 2	
8	F	60–70	23.2	NL	G 2	
9	F	60–70	28.9	OV	G 2	
10	M	55–65	25.8	OV	NL	
11	M	60–65	28.9	OV	G 1	
12	M	30–35	27.5	OV	NL	
13	F	50–55	31.8	OB	G 1	
14	F	30–35	18.5	NL	HNL	
15	F	25–30	22.9	NL	NL	
16	M	30–35	20.3	NL	G 1	
17	F	25–30	28.2	OV	HNL	Lisinopril/Metformin
18	M	60–65	29.6	OV	G 1	
19	F	40–45	25.4	OV	G1	
20	F	45–50	26.7	OV	G1	
Mean ± SD		52.2 ± 17	27.2 ± 4	8 = OB, 5 = NL, 7 = OV	4 = NL, 4 = HNL, 7 = G1, 5 = G 2	

*Notes*: Characteristics of the participant for study B; sex (M = male, F = female), age range in years, body mass index (BMI), BMI Status; BMI normal weight BMI 18.5–24.9 (NL), overweight 25–22.9 (OV) and obese 30 or more (OB). The initial blood pressure status (HTN) at the beginning of the study was classified according to the 2021 European guidelines (Stergiou et al., 2021) Normal optimal BP (NL < 130/85 mmHg), High‐normal BP (HNL, 130–139/85–89 mmHg), Hypertension grade 1 (G1,140–159/90–99 mmHg), Hypertension grade 2 and 3 (G2‐3, ≥160/100 mmHg) and medications used at the time of enrollment.

### 2.3 Jogging device (JD)

JD has been described in previous publications (Adams et al., 2018; Adams et al., 2020; Sackner & Adams, 2017; Sackner et al., 2019). It incorporates a microprocessor controlled DC motor, which produces movements of foot pedals placed within a chassis to repetitively tap against a semi‐rigid surface for effortless simulation of locomotion while the subject is seated or lying in bed. Each time the moving foot pedals hit the bumper, a small pulse is added to the circulation as a function of the pedal speed, which in the current study was set to ~190 steps in place (Sackner et al., 2019). The latter rate is close to the intermittent tap range between 220 and 290 taps/min of voluntary fidgeting that improve endothelial dysfunction, as reflected by increased flow‐mediated vasodilation (Morishima et al., 2016).

### 2.4 Procedures

Study subjects provided their informed consent and were asked to return on a separate day between 8 and 10 am. They were asked not to drink coffee and remain nothing by mouth (NPO) since midnight the previous day of the study. Study A (Acute effects of JD on blood pressure and DN position); On day 1 of the study, the subject was randomized to receive 30 min of JD or CONT using a random choice computer generator [https://www.gigacalculator.com/randomizers/random‐choice‐generator.php]. The subjects were seated in a padded chair and a continuous non‐invasive arterial pressure monitoring device (CNAP, CNSystems, Medizintechnik AG, Graz, Austria), a commercially available system composed of CNAP Monitor 500, the CNAP double finger cuff and the CNAP controller, which was attached to the subject's right forearm. CNAP is based on the principle of vascular unloading technique; the device was placed on the right arm. An infrared finger clip photoplethysmograph (MLT1020FC, ADI instruments, Colorado Springs, CO 80906) was placed on the contralateral index finger. The data acquisition of the CNAP device and the pulse waveform started after 10 min of the subject sitting quietly in a dim light room. The JD was used at (f = 190 steps in place) in seated posture and continued for 30 min; the CONT condition involved sitting in the same location with feet on the JD also for 30 min, but the latter was not turned on. Subjects randomized to JD on day 1, received the CONT intervention on day 3, and those randomized to CONT on day 1 received JD on day 3, therefore, each subject served as their own control (Figure S1). In Study B (Effects of 7 day use of JD on blood pressure and DN position); on day 1 of the study, subjects were also seated in a padded chair and continuous non‐invasive arterial pressure and plethysmographic pulse waveform were measured in the same manner as previously described. The subjects were instructed on home use of JD consisting of at least three times per day for 30 minutes duration (approximately 190 pedal steps in place per minute, more than 10,000 pedal steps in place per day in 1 h). To verify compliance with JD use, they were asked to take pictures of the JD monitoring screen daily with a loaned iPhone and to deliver the iPhone to the study coordinator. To replicate real‐world behavior, subjects were told to continue their current schedule, diet, and physical activity. Subjects returned JD after 7 days (JD7), continuous non‐invasive arterial pressure and plethysmographic pulse waveform were measured as previously described, and the measurements were repeated 48 and 72 h (REC 48, REC 72) after cessation of JD (Figure S3). Neither of the studies could be blinded since the use of the JD device is obvious.

### 2.5 Statistical analysis

Data analysis was performed using Statistica (Statsoft, Tulsa, Ok). Comparison between groups and time for changes in blood pressure, heart rate, and DN were performed using a two‐way repeated measures analysis of variance (ANOVA), with post hoc analysis using Tukey honestly significant difference (HSD). The sample size calculation for Study A, was performed using our previously published data; for a 100% increase in a/b with a type I error of 0.05, and a power of 95%, the required sample size is 18 subjects. A post hoc power analysis using the data from the current study based on a/b with the probability of a type I error of 0.05, is 98.3%. The size of the effect of JD on a/b was calculated using Cohen's d. (Hutcheson & Griffith, 1991) Data are expressed as mean and standard error of the mean (SEM) with statistical significance at p < 0.01, unless otherwise indicated.

### 2.6 Dicrotic notch data processing

Data were continuously collected from the CNAP device and finger plethysmograph on an eight channel Power Lab (ADI instruments, Colorado Springs, CO 80906) connected to a PC, at a sampling rate of 1000 points per second. The method to analyze blood pressure data was previously reported (Sackner et al., 2019). The digital pulse waveform of the plethysmograph was analyzed using Lab Chart 7 software (ADI instruments, Colorado Springs, CO 80906). The change in the position of the DN or wave is computed by measuring the amplitude of the digital pulse wave (a) divided by the height of the dicrotic notch or wave above the end diastolic level (b), the ratio of a/b. In this study, the DN instead of the dichroic wave was utilized to compute the a/b ratio. To aid in the detection of the DN, the second derivative of the pulse wave and the peak of the largest upward deflection in diastole were taken as the point of DN. Twenty pulse waveforms per time interval were averaged to obtain the average positions of a and b. Five‐minute epochs were analyzed for blood pressure and a/b measurements. In Study A, measurements were obtained at baseline (BL) and after 5, 10, 20, 30, 35, 60 min (T5,T10, T20, T30,) and 5 and 60 min after completion of JD or CONT (Recovery, REC 5, 60) (Figure S1). In Study B, measurements of blood pressure and a/b were obtained at BL (prior to starting JD), after 7 days of JD (JD7) and after 48 and 72 h of discontinuation of JD (Recovery, REC 48, 72) (Figure S3).

## 3 RESULTS

### 3.1 Study A: Acute effects of JD on blood pressure and position of DN

**Heart rate.** Heart rate remained unchanged during control and the use of JD.

**FIGURE 1:**
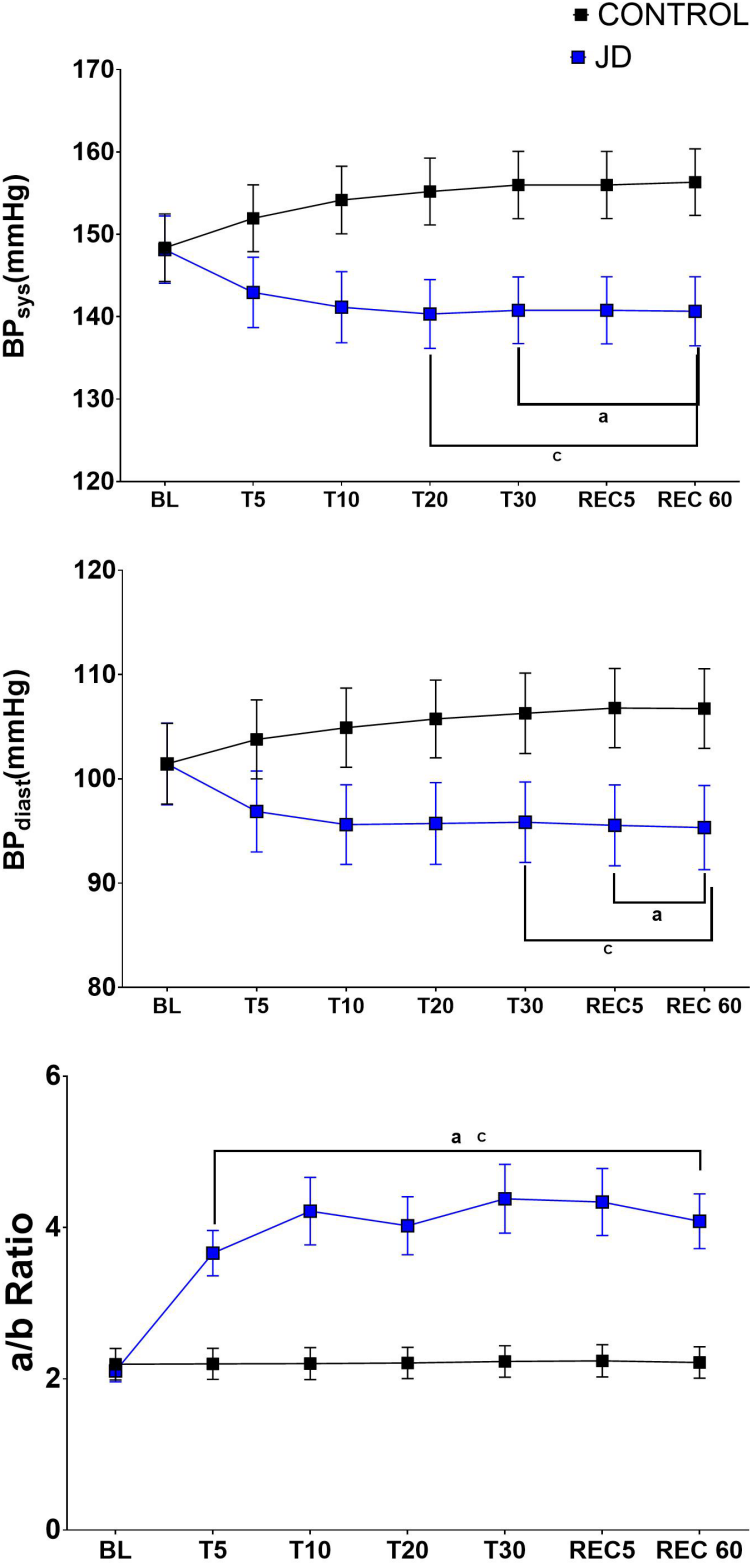
The Acute Effects of JD on Blood Pressure and Dicrotic Notch Position (a/b). Effects of JD on systolic blood pressure (mmHg) (Panel A), diastolic blood pressure (mmHg) (Panel B), and descent of the dicrotic notch (a/b). Data are mean, and standard error of the mean. There was a statistically significant decrease compared to BL in both BPs, BPd and a significant increase in a / b (^a^ *p* < 0.001 JD vs. BL; ^b^ *p* <0.001 CONT vs. BL; ^c^ *p* < 0.001 CONT vs. JD).

**Blood pressure**. In Study A, there was a significant decrease in both systolic and diastolic blood pressures (BPs, BPd) from BL during and after JD use. At its nadir, systolic blood pressure decreased by 21 mmHg in REC5 and similarly diastolic blood pressure also decreased by 19 mmHg in REC5. Systolic and diastolic blood pressures decreased significantly from BL from T30 to 60 min after discontinuation of JD. On average, systolic BP decreased approximately 12% and diastolic 17% from BL values (Table 3 and Figure 1). In contrast, during CONT condition both systolic and diastolic blood pressure increased on average by 4 mmHg. There were no statistically significant correlations between age and BPs, BPd or a/b ratio.

**TABLE 3:** Hemodynamics and a/b for Study A; the acute effects of JD on blood pressure and DN position

	BL		T5		T10		T20		T30		REC 5		REC60	
	*CONT*	*JD*	*CONT*	*JD*	*CONT*	*JD*	*CONT*	*JD*	*CONT*	*JD*	*CONT*	*JD*	*CONT*	*JD*
Heart Rate (BPM)	68.9 (2.3)	69.5 (2.3)	69.1 (2.0)	68.7 (2.2)	70.3 (2.5)	69.0 (2.3)	72.1 (2.7)	71.1 (2.1)	69.8 (2.5)	69.1 (2.0)	70.1 (2.7)	69.6 (2.1)	68.9 (2.9)	69.8 (2.1)
BPs (mmHg)	148 (4.1)	148 (4.1)	150 (4.1)	131 (4.3)	149 (4.1)	131 (4.3)	151 (4.1)	130 (4.1)^c^	152 (4.1)	128 (3.7)^c,a^	153 (4.1)	127 (3.9)^c,a^	153 (4.3)	128 (3.5)^c,a^
BPd (mmHg)	101 (3.9)	101 (3.9)	101 (3.9)	89 (3.5)	103 (3.9)	86 (3.5)	103 (3.9)	86 (3.5)	104 (4.1)	84 (3.5)^c^	105 (3.9)	82 (3.3)^c,a^	106 (3.9)	84 (3.3)^c,a^
a/b	2.2 (0.2)	2.1 (0.1)	2.2 (0.2)	3.7 (0.3)^a^	2.2 (0.2)	4.2 (0.4)^c,a^	2.2 (0.2)	4.0 (0.4)^c,a^	2.2 (0.2)	4.4 (0.4)^c,a^	2.2 (0.2)	4.3 (0.4)^c,a^	2.2 (0.2)	4.1 (0.4)^c,a^
Percent Change Baseline (%)														
BPs	0.0	0.0	0.9 (0.2)	−11.8 (0.8)^c,a^	0.6 (0.2)	−12.1 (0.7)^c,a^	1.7 (0.3)	−12.7 (0.3)^c,a^	2.5 (0.4)	−13.4 (0.7)^c,a^	2.8 (0.4)^b^	−14.3 (0.9)^c,a^	3.4 (0.3)^b^	−13.7 (0.6)^c,a^
BPd	0.0	0.0	−0.1 (0.2)	−12.1 (1.3)^c,a^	1.3 (0.1)	−14.5 (1.4)^c,a^	2.1 (0.4)	−17.7 (2.5)^c,a^	2.9 (0.4)	−16.6 (1.4)^c,a^	3.6 (0.4)	−18.6 (1.6)^c,a^	4.2 (0.4)	−17.3 (1.5)^c,a^
a/b	0.0	0.0	0.5 (0.8)	81.1 (14.9)^c,a^	0.4 (1.3)	102.8 (15.3)^c,a^	1.1 (1.3)	96.2 (14.4)^c,a^	2.0 (1.2)	109.1 (14.2)^c,a^	1.0 (1.9)	106.5 (13.4)^c,a^	1.2 (1.1)	98.4 (14.2)^c,a^

*Notes*: Hemodynamic variables; heart rate (BPM), systolic and diastolic blood pressure (BPs, BPd) and a/b. During the CONT (Control) condition, the subject is in seated posture with the feet on the JD, but the latter was not turned on. In the JD group, JD was used under the same conditions beginning after a baseline measurement (BL) for 30 min, T30 (Figure S1). Statistical comparisons between the JD and CONT groups were made at times 5, 10, 20, 30 min (T5, T10, T20, T30), and 5 and 60 min after discontinuation of JD or CONT (REC 5, REC 60). Data expressed as mean and standard error of the mean (±SEM). There was a statistically significant decrease in absolute and percentage changes compared to BL in both BPs, BPd and a significant increase in a / b (^a^ *p* < 0.001 JD vs BL; ^b^ *p* <0.001 CONT vs BL; ^c^ *p* < 0.001 CONT vs JD).

In Study A, a/b increased significantly from BL during JD use and up to 60 min after discontinuation of JD (REC 60) (Table 3 and Figure 1). Seventy‐nine percent of all participants had an increase in a/b from BL to REC 60 of greater than 50% (Table S1 and Figure S5 panel A). During the control condition, a/b was unchanged over time.

There were no significant differences in systolic, diastolic pressures, a/b, or change from baseline in these, between male or female participants. Furthermore, when the study population was arbitrarily stratified into younger (<60 years age) or older (>60 years age), there was also no difference in the effects of JD on blood pressure or a/b between these two age groups.

### 3.2 Effects of 7‐day use of JD on blood pressure and DN position

**Heart rate.** Heart rate remained unchanged from BL after 7 days of JD and REC 48 and 72 h.

**FIGURE 2:**
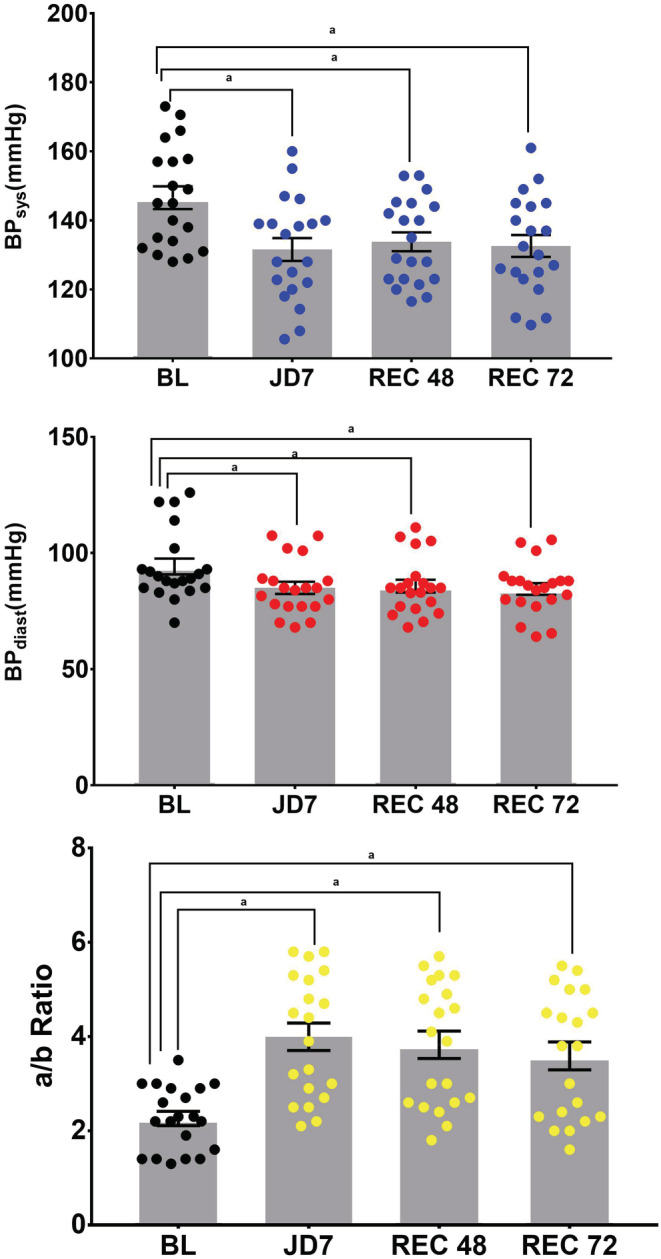
The Effects of 7 days of JD on Systolic and Diastolic Blood Pressure and Dicrotic Notch Descent (a/b). Effects of 7 days of JD use on systolic blood pressure (mmHg) (Panel A), diastolic blood pressure (mmHg) (panel B) and the descent of the dicrotic notch (a/b), at baseline (BL), and 7 days of JD (JD7) and 48 and 72 h after discontinuation of JD (REC 48, REC 72) Data are individual subjects, mean and standard error of the mean. There is a statistically significant effect of the JD at all time points compared to BL. ^a^ *p* < 0.001 BL vs. JD7, REC 48, REC72.

**Blood pressure.** In Study B, BPs decreased from BL on average 15 mmHg after 7 days of JD (JD7) and remained lower than BL by 14 mmHg, 72 h after discontinuation of JD. Similarly, BPd also decreased from BL on average of 9 mmHg at JD7 and remained lower than BL by 9 mmHg, 72 h after discontinuation of JD (REC 72) (Table 4 and Figure 2).

**TABLE 4:** Hemodynamics and a/b for Study B; effects of 7 day use of JD on blood pressure and DN position group

	BL	JD7	REC 48	REC 72
Heart rate (BPM/)	64 (1.8)	64 (2)	64 (1.8)	63 (1.8)
BPs (mmHg)	147 (3.3)	132 (3.3)^a^	134 (2.7)^a^	133 (3.1)^a^
BPd (mmHg)	94 (3.3)	85 (2.7)^a^	86 (2.7)^a^	85 (2.4)^a^
a/b	2.3 (0.7)	4.0 (1.3)^a^	3.8 (1.3)^a^	3.6 (1.3)^a^
Percent change baseline (%)				
BPs	0	−10.3 (0.9)^a^	−8.6 (0.6)^a^	−9.5 (0.9)^a^
BPd	0	−9.2 (1.4)^a^	−8.5 (1.6)^a^	−9.7 (1.7)^a^
a/b	0	82.4 (11.1)^a^	73.9 (11.7)^a^	63.2 (12.5)^a^

*Notes*: In Study B, Hemodynamic variables; heart rate (BPM), blood pressure systolic and diastolic (BPs, BPd) and a/b. At baseline (BL) and after 7 days of daily use of JD (at least 3 times per day for 30 min, JD7), and 48 and 72 h after discontinuation of JD (REC48, REC72) (Figure S3). Statistical comparison was performed between BL and JD7, REC 48, and REC 72. Data expressed as mean and standard error of the mean (±SEM). Compared to BL there was a statistically significant decrease in both absolute and percent change in both BPs, BPd and a significant increase in a / b (^a^ *p* < 0.001 BL vs. JD7, REC 48, REC 72).

**a/b ratio.** In Study B, a/b significantly increased from BL after 7 days of JD by 82%, and remained increased by 63% at REC 72 (Table 4 and Figure 2).

### 3.3 Effect size of JD on a/b

In Study A, 79% of the study participants had a greater than 50% change in a/b after 30 min of JD use (Table S1, S2 and Figure S5 panel A). In Study B, 85% of the study participants had a greater than 50% change in a/b after 7 days of JD (JD7) (Table S1, S3 and Figure S5 panel B). Additionally, the effect size of JD on a/b for both studies was calculated at all study periods using Cohen's d, with values that ranged from 1.17 to 1.63. A value greater than or equal to 0.8 denotes a large effect size (Table S4).

## 4 DISCUSSION

In large part, the beneficial health effects of JD relate to its increase in pulsatile endothelial shear stress (friction) as found for its predicate device, the motion platform (Whole Body Periodic Acceleration,WBPA aka pGz) (Uryash et al., 2009). JD was based on pioneering experiments in a perfused isolated blood vessel by Hutcheson and Griffith (Hutcheson & Griffith, 1991). They found that NO was released from the endothelium of perfused blood vessels from pulses delivered of 2 mmHg with a peak response between 250 and 360 pulses per minute. We have confirmed observations in isolated blood vessel experiments (Uryash et al., 2009; Uryash et al., 2015; Wu et al., 2012) and whole animal experiments (Hoeksel et al., 1997; Uryash et al., 2009; Weinberg, Habens, Kengatharan, Barnes, Matz, Anggard, & Carrier, 2001) that pulsatile shear stress on the vascular endothelium induces NO production by endothelial nitric oxide synthase (eNOS). The increase in NO explained vasodilation (Uryash et al., 2015). We have also shown that pulsatile shear stress upregulates eNOS and induces phosphorylation and activation of eNOS (Uryash et al., 2015; Wu et al., 2012). The use of the a/b ratio as a surrogate for changes in NO has been shown by others (Hoeksel et al., 1997; Klemsdal et al., 1994; Millasseau, 2003; Nier et al., 2001; Nier et al., 2008; Weinberg, Habens, Kengatharan, Barnes, Matz, Anggard, & Carrier, 2001) and in human subjects using the predicate device WBPA (Fujita et al., 2005; Sackner et al., 2005a; Sackner et al., 2005b). In the current study, we show that JD increases the a/b ratio, and thus NO, within the first 5 min of JD operation and continues for at least 60 min after discontinuation of JD. The characteristics of the response curve over time suggest that an acute increase in NO (within 5 min) is followed by sustained release, which persists for at least 60 min after discontinuation. Furthermore, we have confirmed that continued use of JD for 7 days (at least three times a day for 30 min) also increases NO compared to baseline values for at least 72 h after stopping JD. These data are consistent with our previously reported studies in mice using the WBPA predicate device. The latter showed that a single one‐hour session of WBPA increases eNOS phosphorylation, and daily use of WBPA induces up‐regulation of eNOS mRNA (Uryash et al., 2015).

The mechanics of JD are more akin to passive leg movement than to external compression or active exercise. Investigators have used passive leg movement as a means of increasing vascular shear stress and have shown a nearly three‐fold increase in NO‐dependent blood flow (Broxterman et al., 2017; Groot et al., 2015; Mortensen et al., 2012), along with increased genomic expression of eNOS (Hellsten et al., 2008; Hoier et al., 2010; Trinity et al., 2015) and have further advocated for the use of passive leg movement as an approach to assess NO‐mediated vascular function (Trinity et al., 2012). Experiments carried out by Trinity et al. using passive leg movement (PLM, 60 cycles/min for 1 min) or single PLM (sPLM, 1 s) in young healthy volunteer males showed that the predominant endothelial‐dependent pathway (60%–80%) involved in the hyperemic response to both PLM and sPLM is NO‐mediated. Furthermore, pharmacological blockade of other endothelial dependent pathways such as prostaglandins (PG) and endothelial‐derived hyperpolarizing factor (EDHF) does not further reduce the hyperemic response of PLM compared to NO inhibition alone, and therefore endothelial independent pathways may be operative in the hyperemic response (Trinity et al., 2021).

Genetic and epidemiological studies indicate that deficiencies in NO synthesis and bioavailability derived from eNOS are central to the pathogenesis of hypertension, reduced cardiac and endothelial cell function, and metabolic disorders (Emdin et al., 2018; Erdmann et al., 2013; Johnstone et al., 1993; Monti et al., 2003; Oemar et al., 1998; Petrie et al., 1996; Sansbury et al., 2012). Furthermore, a genetic predisposition to enhanced NO signaling offers protection against cardiovascular diseases. As reviewed by Lundberg et al. (Lundberg et al., 2015) interventions that restore NO have been associated with improved cardiovascular and metabolic health. Therefore, the clinical implications of our findings are important, as we can achieve a noninvasive, nonpharmacological increase in NO, which, in addition to improved vascular hemodynamics, can also have a multitude of beneficial effects on the cardiovascular and central nervous system (Ashor et al., 2015; da Luz & Latini, 2020; Gertz et al., 2006; Katusic & Austin, 2016; Marino et al., 2021; Paillard et al., 2015; Zhang & Gao, 2021), as well as being integral to intracellular function, adaptation and signaling (Daiber et al., 2019; Forstermann & Li, 2011; Forstermann & Sessa, 2012; Gantner et al., 2020; Ghimire et al., 2017; Hsieh et al., 2014; Muzorewa et al., 2021; Poderoso et al., 2019; Vanhoutte, 2018).

### 4.1 Cardioprotective effects of increasing NO

In the cardiovascular system, previous studies have shown the beneficial effects of JD on sedentary‐induced elevated blood pressure. In normotensive and hypertensive adult subjects during physical inactivity in seated and supine postures, systolic blood pressure (BP) increased by 7.5 and 10.4 mmHg, respectively. JD decreased seated and supine BPs by 8.4 and 11.2 mmHg, respectively. The effect persisted for at least 1 h after discontinuation of JD (Sackner et al., 2019). Similarly, in the current study in seated posture, a 5 mmHg increase in BPs, was observed and JD decreased BPs by 20 mmHg. Furthermore, longer‐term use of JD (7 days) also decreased BPs by 14 mmHg, and this effect persists for at least 72 h after discontinuation of JD (REC72). The effects of decreasing BP by adding pulses to the circulation are largely induced by NO. In anesthetized pigs, the addition of pulsations (180 per min) using WBPA decreases the mean femoral blood pressure from 94 mmHg at baseline to 80 mmHg after 30 minutes, with the corresponding mean pulmonary artery pressure decreasing from 12 mmHg to 10 mmHg. This effect is attenuated by L‐NAME, a nitric oxide synthase inhibitor, which demonstrates that the vasodilator properties of pulsatile shear stress in the endothelium are consistent with an increase in NO (Adams et al., 2000). In human subjects, various modes of exercise have been shown to induce a post‐exercise decrease in blood pressure in both hypertensive and nonhypertensive subjects (Borjesson et al., 2016; Cornelissen & Smart, 2013) and increase NO (Ashor et al., 2015; Goto et al., 2007). Mechanisms for shear stress and increased NO induced by exercise have been thoroughly reviewed (Gielen et al., 2010; Green et al., 2004; Schuler et al., 2013) and confirmed at the cell level the use of simulated exercise‐induced endothelial wall shear stress (Wang et al., 2019).

## 5 STUDY LIMITATIONS

The current study was designed as a noninvasive study and therefore was not designed to invasively (blood or tissue) compare the biochemical production of NO, and no direct method was used to determine the increase in NO induced by JD. Thus, it is possible that a discreet dose response to JD may exist but changes in the descent of DN are not sensitive enough to detect such small changes. The descent of the DN or wave on the descending limb of the arterial pulse waveform is a surrogate measure of NO and detects the overall vascular effect. The data showed a sustained and time‐dependent increase in a/b ratio, a surrogate marker for NO. We also did not define a priori a specific study population or composition, as our goal was to test the effects of JD on NO in both genders and subjects likely to use JD. Additionally, we did not investigate the carry‐over effect of JD on BP or a/b beyond 72 h after discontinuation of JD. The data further confirms our previous observations on the acute and longer‐term (7 days) effects of JD in decreasing both BPs and BPd.

## 6 CONCLUSION

The application of JD altered the descent of the dicrotic notch (a/b) consistent with increases in NO. The effect appears within the first 5 min of JD use and remains at least during the 30 min of JD use and 60 min after use, with an increase in NO lasting for at least 72 h after stopping a 7‐day JD use. In addition to the previously reported beneficial health effects of JD on diabetes, hypertension, sedentary lifestyle, mobility, and improved heart rate variability, JD is poised to be a simple nonpharmacologic and noninvasive strategy to harness the beneficial effects of increasing endogenous NO, particularly in subjects who cannot exercise.

## AUTHORS' CONTRIBUTIONS

JAA study design, data analysis and manuscript writing. MAS study design and 1st draft of manuscript. JRL study design, data acquisition, analysis. VN manuscript writing. ZC manuscript writing. HI manuscript writing.

## Supporting information


Data S1

## References

[phy215418-bib-0001] Adams, J. A. , Banderas, V. , Lopez, J. R. , & Sackner, M. A. (2020). Portable gentle jogger improves glycemic indices in type 2 diabetic and healthy subjects living at home: A pilot study. Journal of Diabetes Research, 2020, 8317973. 32215273 10.1155/2020/8317973 PMC7081036

[phy215418-bib-0002] Adams, J. A. , Lopez, J. R. , Banderas, V. , & Sackner, M. A. (2021). A single arm trial using passive simulated jogging for blunting acute hyperglycemia. Scientific Reports, 11, 6437. 33742027 10.1038/s41598-021-85579-7 PMC7979828

[phy215418-bib-0003] Adams, J. A. , Mangino, M. J. , Bassuk, J. , & Sackner, M. A. (2000). Hemodynamic effects of periodic G(z) acceleration in meconium aspiration in pigs. Journal of Applied Physiology (1985), 89, 2447–2452. 10.1152/jappl.2000.89.6.2447 11090601

[phy215418-bib-0004] Adams, J. A. , Patel, S. , Lopez, J. R. , & Sackner, M. A. (2018). The effects of passive simulated jogging on short‐term heart rate variability in a heterogeneous group of human subjects. Journal of Sports Medicine (Hindawi Publ Corp), 4340925, 2018. 10.1155/2018/4340925 PMC6191954 30402499

[phy215418-bib-0005] Adams, V. , & Linke, A. (2019). Impact of exercise training on cardiovascular disease and risk. Biochimica et Biophysica Acta ‐ Molecular Basis of Disease, 728–734, 2019. 10.1016/j.bbadis.2018.08.019 30837069

[phy215418-bib-0006] Ashor, A. W. , Lara, J. , Siervo, M. , Celis‐Morales, C. , Oggioni, C. , Jakovljevic, D. G. , & Mathers, J. C. (2015). Exercise modalities and endothelial function: A systematic review and dose‐response meta‐analysis of randomized controlled trials. Sports Medicine, 45, 279–296. 25281334 10.1007/s40279-014-0272-9

[phy215418-bib-0007] Bahadoran, Z. , Carlstrom, M. , Mirmiran, P. , & Ghasemi, A. (2020). Nitric oxide: To be or not to be an endocrine hormone? Acta Physiologica (Oxford, England), 229, e13443. 10.1111/apha.13443 31944587

[phy215418-bib-0008] Balligand, J. L. , Feron, O. , & Dessy, C. (2009). eNOS activation by physical forces: From short‐term regulation of contraction to chronic remodeling of cardiovascular tissues. Physiological Reviews, 89, 481–534. 19342613 10.1152/physrev.00042.2007

[phy215418-bib-0009] Borjesson, M. , Onerup, A. , Lundqvist, S. , & Dahlof, B. (2016). Physical activity and exercise lower blood pressure in individuals with hypertension: Narrative review of 27 RCTs. British Journal of Sports Medicine, 50, 356–361. 26787705 10.1136/bjsports-2015-095786

[phy215418-bib-0010] Broxterman, R. M. , Trinity, J. D. , Gifford, J. R. , Kwon, O. S. , Kithas, A. C. , Hydren, J. R. , Nelson, A. D. , Morgan, D. E. , Jessop, J. E. , Bledsoe, A. D. , & Richardson, R. S. (2017). Single passive leg movement assessment of vascular function: Contribution of nitric oxide. Journal of Applied Physiology (1985), 123, 1468–1476. 10.1152/japplphysiol.00533.2017 PMC5814686 28860173

[phy215418-bib-0011] Cornelissen, V. A. , & Smart, N. A. (2013). Exercise training for blood pressure: A systematic review and meta‐analysis. Journal of the American Heart Association, 2, e004473. 23525435 10.1161/JAHA.112.004473 PMC3603230

[phy215418-bib-0012] da Luz, S. D. , & Latini, A. (2020). Exercise‐induced immune system response: Anti‐inflammatory status on peripheral and central organs. Biochimica et Biophysica Acta ‐ Molecular Basis of Disease, 165823, 165823. 10.1016/j.bbadis.2020.165823 PMC7188661 32360589

[phy215418-bib-0013] Daiber, A. , Xia, N. , Steven, S. , Oelze, M. , Hanf, A. , Kroller‐Schon, S. , Munzel, T. , & Li, H. (2019). New therapeutic implications of endothelial nitric oxide synthase (eNOS) function/dysfunction in cardiovascular disease. International Journal of Molecular Sciences, 20, 187. 10.3390/ijms20010187 PMC6337296 30621010

[phy215418-bib-0014] Emdin, C. A. , Khera, A. V. , Klarin, D. , Natarajan, P. , Zekavat, S. M. , Nomura, A. , Haas, M. , Aragam, K. , Ardissino, D. , Wilson, J. G. , Schunkert, H. , McPherson, R. , Watkins, H. , Elosua, R. , Bown, M. J. , Samani, N. J. , Baber, U. , Erdmann, J. , Gormley, P. , … Kathiresan, S. (2018). Phenotypic consequences of a genetic predisposition to enhanced nitric oxide signaling. Circulation, 137, 222–232. 28982690 10.1161/CIRCULATIONAHA.117.028021 PMC5771958

[phy215418-bib-0015] Erdmann, J. , Stark, K. , Esslinger, U. B. , Rumpf, P. M. , Koesling, D. , de Wit, C. , Kaiser, F. J. , Braunholz, D. , Medack, A. , Fischer, M. , Zimmermann, M. E. , Tennstedt, S. , Graf, E. , Eck, S. , Aherrahrou, Z. , Nahrstaedt, J. , Willenborg, C. , Bruse, P. , Braenne, I. , … Schunkert, H. (2013). Dysfunctional nitric oxide signalling increases risk of myocardial infarction. Nature, 504, 432–436. 24213632 10.1038/nature12722

[phy215418-bib-0016] Farah, C. , Michel, L. Y. M. , & Balligand, J.‐L. (2018). Nitric oxide signalling in cardiovascular health and disease. Nature Reviews Cardiology, 15, 292–316. 29388567 10.1038/nrcardio.2017.224

[phy215418-bib-0017] Forstermann, U. , & Li, H. (2011). Therapeutic effect of enhancing endothelial nitric oxide synthase (eNOS) expression and preventing eNOS uncoupling. British Journal of Pharmacology, 164, 213–223. 21198553 10.1111/j.1476-5381.2010.01196.x PMC3174401

[phy215418-bib-0018] Forstermann, U. , & Sessa, W. C. (2012). Nitric oxide synthases: Regulation and function. European Heart Journal, 33, 829–837 837a‐837d. 21890489 10.1093/eurheartj/ehr304 PMC3345541

[phy215418-bib-0019] Fujita, M. , Tambara, K. , Ikemoto, M. , Sakamoto, S. , Ogai, A. , Kitakaze, M. , & Sackner, M. (2005). Periodic acceleration enhances release of nitric oxide in healthy adults. International Journal of Angiology, 14, 11–14.

[phy215418-bib-0020] Gantner, B. N. , LaFond, K. M. , & Bonini, M. G. (2020). Nitric oxide in cellular adaptation and disease. Redox Biology, 34, 101550. 32438317 10.1016/j.redox.2020.101550 PMC7235643

[phy215418-bib-0021] Gertz, K. , Priller, J. , Kronenberg, G. , Fink, K. B. , Winter, B. , Schrock, H. , Ji, S. , Milosevic, M. , Harms, C. , Bohm, M. , Dirnagl, U. , Laufs, U. , & Endres, M. (2006). Physical activity improves long‐term stroke outcome via endothelial nitric oxide synthase‐dependent augmentation of neovascularization and cerebral blood flow. Circulation Research, 99, 1132–1140. 17038638 10.1161/01.RES.0000250175.14861.77

[phy215418-bib-0022] Ghimire, K. , Altmann, H. M. , Straub, A. C. , & Isenberg, J. S. (2017). Nitric oxide: What's new to NO? American Journal of Physiology. Cell Physiology, 312, C254–C262. 27974299 10.1152/ajpcell.00315.2016 PMC5401944

[phy215418-bib-0023] Gielen, S. , Schuler, G. , & Adams, V. (2010). Cardiovascular effects of exercise training: molecular mechanisms. Circulation, 122, 1221–1238. 20855669 10.1161/CIRCULATIONAHA.110.939959

[phy215418-bib-0024] Goto, C. , Nishioka, K. , Umemura, T. , Jitsuiki, D. , Sakagutchi, A. , Kawamura, M. , Chayama, K. , Yoshizumi, M. , & Higashi, Y. (2007). Acute moderate‐intensity exercise induces vasodilation through an increase in nitric oxide bioavailiability in humans. American Journal of Hypertension, 20, 825–830. 17679027 10.1016/j.amjhyper.2007.02.014

[phy215418-bib-0025] Green, D. J. , Maiorana, A. , O'Driscoll, G. , & Taylor, R. (2004). Effect of exercise training on endothelium‐derived nitric oxide function in humans. The Journal of Physiology, 561, 1–25. 15375191 10.1113/jphysiol.2004.068197 PMC1665322

[phy215418-bib-0026] Groot, H. J. , Trinity, J. D. , Layec, G. , Rossman, M. J. , Ives, S. J. , Morgan, D. E. , Bledsoe, A. , & Richardson, R. S. (2015). The role of nitric oxide in passive leg movement‐induced vasodilatation with age: Insight from alterations in femoral perfusion pressure. The Journal of Physiology, 593, 3917–3928. 26108562 10.1113/JP270195 PMC4575577

[phy215418-bib-0027] Hambrecht, R. , Adams, V. , Erbs, S. , Linke, A. , Krankel, N. , Shu, Y. , Baither, Y. , Gielen, S. , Thiele, H. , Gummert, J. F. , Mohr, F. W. , & Schuler, G. (2003). Regular physical activity improves endothelial function in patients with coronary artery disease by increasing phosphorylation of endothelial nitric oxide synthase. Circulation, 107, 3152–3158. 12810615 10.1161/01.CIR.0000074229.93804.5C

[phy215418-bib-0028] Hellsten, Y. , Rufener, N. , Nielsen, J. J. , Hoier, B. , Krustrup, P. , & Bangsbo, J. (2008). Passive leg movement enhances interstitial VEGF protein, endothelial cell proliferation, and eNOS mRNA content in human skeletal muscle. American Journal of Physiology. Regulatory, Integrative and Comparative Physiology, 294, R975–R982. 18094062 10.1152/ajpregu.00677.2007

[phy215418-bib-0029] Hoeksel, S. A. , Jansen, J. R. , Blom, J. A. , & Schreuder, J. J. (1997). Detection of dicrotic notch in arterial pressure signals. Journal of Clinical Monitoring, 13, 309–316. 9338845 10.1023/a:1007414906294

[phy215418-bib-0030] Hoier, B. , Rufener, N. , Bojsen‐Moller, J. , Bangsbo, J. , & Hellsten, Y. (2010). The effect of passive movement training on angiogenic factors and capillary growth in human skeletal muscle. The Journal of Physiology, 588, 3833–3845. 20693292 10.1113/jphysiol.2010.190439 PMC2998230

[phy215418-bib-0031] Hsieh, H. J. , Liu, C. A. , Huang, B. , Tseng, A. H. , & Wang, D. L. (2014). Shear‐induced endothelial mechanotransduction: The interplay between reactive oxygen species (ROS) and nitric oxide (NO) and the pathophysiological implications. Journal of Biomedical Science, 21, 3. 24410814 10.1186/1423-0127-21-3 PMC3898375

[phy215418-bib-0032] Hutcheson, I. R. , & Griffith, T. M. (1991). Release of endothelium‐derived relaxing factor is modulated both by frequency and amplitude of pulsatile flow. The American Journal of Physiology, 261, H257–H262. 1858928 10.1152/ajpheart.1991.261.1.H257

[phy215418-bib-0033] Johnstone, M. T. , Creager, S. J. , Scales, K. M. , Cusco, J. A. , Lee, B. K. , & Creager, M. A. (1993). Impaired endothelium‐dependent vasodilation in patients with insulin‐dependent diabetes mellitus. Circulation, 88, 2510–2516. 8080489 10.1161/01.cir.88.6.2510

[phy215418-bib-0034] Katusic, Z. S. , & Austin, S. A. (2016). Neurovascular protective function of endothelial nitric oxide‐ recent advances. Circulation Journal, 80, 1499–1503. 27238834 10.1253/circj.CJ-16-0423

[phy215418-bib-0035] Klemsdal, T. O. , Andersson, T. L. , Matz, J. , Ferns, G. A. , Gjesdal, K. , & Anggard, E. E. (1994). Vitamin E restores endothelium dependent vasodilatation in cholesterol fed rabbits: In vivo measurements by photoplethysmography. Cardiovascular Research, 28, 1397–1402. 7954652 10.1093/cvr/28.9.1397

[phy215418-bib-0036] Lundberg, J. O. , Gladwin, M. T. , & Weitzberg, E. (2015). Strategies to increase nitric oxide signalling in cardiovascular disease. Nature Reviews. Drug Discovery, 14, 623–641. 26265312 10.1038/nrd4623

[phy215418-bib-0037] Marino, F. , Scalise, M. , Cianflone, E. , Salerno, L. , Cappetta, D. , Salerno, N. , De Angelis, A. , Torella, D. , & Urbanek, K. (2021). Physical exercise and cardiac repair: The potential role of nitric oxide in boosting stem cell regenerative biology. Antioxidants (Basel), 10, 1002. 34201562 10.3390/antiox10071002 PMC8300666

[phy215418-bib-0038] Millasseau, S. (2003). The vascular impact of aging andvasoactive drugs: Comparison of twodigital volume pulse measurements. American Journal of Hypertension, 16, 467–472. 12799095 10.1016/s0895-7061(03)00569-7

[phy215418-bib-0039] Monti, L. D. , Barlassina, C. , Citterio, L. , Galluccio, E. , Berzuini, C. , Setola, E. , Valsecchi, G. , Lucotti, P. , Pozza, G. , Bernardinelli, L. , Casari, G. , & Piatti, P. (2003). Endothelial nitric oxide synthase polymorphisms are associated with type 2 diabetes and the insulin resistance syndrome. Diabetes, 52, 1270–1275. 12716763 10.2337/diabetes.52.5.1270

[phy215418-bib-0040] Morishima, T. , Restaino, R. M. , Walsh, L. K. , Kanaley, J. A. , Fadel, P. J. , & Padilla, J. (2016). Prolonged sitting‐induced leg endothelial dysfunction is prevented by fidgeting. American Journal of Physiology, 311, H177–H182. 27233765 10.1152/ajpheart.00297.2016 PMC4967200

[phy215418-bib-0041] Mortensen, S. P. , Askew, C. D. , Walker, M. , Nyberg, M. , & Hellsten, Y. (2012). The hyperaemic response to passive leg movement is dependent on nitric oxide: A new tool to evaluate endothelial nitric oxide function. The Journal of Physiology, 590, 4391–4400. 22733658 10.1113/jphysiol.2012.235952 PMC3473293

[phy215418-bib-0042] Muzorewa, T. T. , Buerk, D. G. , Jaron, D. , & Barbee, K. A. (2021). Coordinated regulation of endothelial calcium signaling and shear stress‐induced nitric oxide production by PKCbeta and PKCeta. Cellular Signalling, 87, 110125. 34474112 10.1016/j.cellsig.2021.110125

[phy215418-bib-0043] Nier, B. A. , Carrier, M. J. , Harrington, L. S. , & Weinberg, P. D. (2001). Effects of the nitrergic, adrenergic and cyclo‐oxygenase pathways on pulse waveform in the rabbit. British Journal of Pharmacology, 133, 361.11375252

[phy215418-bib-0044] Nier, B. A. , Harrington, L. S. , Carrier, M. J. , & Weinberg, P. D. (2008). Evidence for a specific influence of the nitrergic pathway on the peripheral pulse waveform in rabbits. Experimental Physiology, 93, 503–512. 18223024 10.1113/expphysiol.2007.041129

[phy215418-bib-0045] Nosarev, A. V. , Smagliy, L. V. , Anfinogenova, Y. , Popov, S. V. , & Kapilevich, L. V. (2014). Exercise and NO production: Relevance and implications in the cardiopulmonary system. Frontiers in Cell and Developmental Biology, 2, 73. 25610830 10.3389/fcell.2014.00073 PMC4285794

[phy215418-bib-0046] Oemar, B. S. , Tschudi, M. R. , Godoy, N. , Brovkovich, V. , Malinski, T. , & Luscher, T. F. (1998). Reduced endothelial nitric oxide synthase expression and production in human atherosclerosis. Circulation, 97, 2494–2498. 9657467 10.1161/01.cir.97.25.2494

[phy215418-bib-0047] Paillard, T. , Rolland, Y. , & de Souto, B. P. (2015). Protective effects of physical exercise in Alzheimer's disease and Parkinson's disease: A narrative review. Journal of Clinical Neurology, 11, 212–219. 26174783 10.3988/jcn.2015.11.3.212 PMC4507374

[phy215418-bib-0048] Park, S. K. , La Salle, D. T. , Cerbie, J. , Cho, J. M. , Bledsoe, A. , Nelson, A. , Morgan, D. E. , Richardson, R. S. , Shiu, Y. T. , Boudina, S. , Trinity, J. D. , & Symons, J. D. (2019). Elevated arterial shear rate increases indexes of endothelial cell autophagy and nitric oxide synthase activation in humans. American Journal of Physiology, 316, H106–H112. 30412436 10.1152/ajpheart.00561.2018 PMC6734082

[phy215418-bib-0049] Petrie, J. R. , Ueda, S. , Webb, D. J. , Elliott, H. L. , & Connell, J. M. (1996). Endothelial nitric oxide production and insulin sensitivity. A physiological link with implications for pathogenesis of cardiovascular disease. Circulation, 93, 1331–1333. 8641020 10.1161/01.cir.93.7.1331

[phy215418-bib-0050] Poderoso, J. J. , Helfenberger, K. , & Poderoso, C. (2019). The effect of nitric oxide on mitochondrial respiration. Nitric Oxide, 88, 61–72. 30999001 10.1016/j.niox.2019.04.005

[phy215418-bib-0051] Rokutanda, T. , Izumiya, Y. , Miura, M. , Fukuda, S. , Shimada, K. , Izumi, Y. , Nakamura, Y. , Araki, S. , Hanatani, S. , Matsubara, J. , Nakamura, T. , Kataoka, K. , Yasuda, O. , Kaikita, K. , Sugiyama, S. , Kim‐Mitsuyama, S. , Yoshikawa, J. , Fujita, M. , Yoshiyama, M. , & Ogawa, H. (2011). Passive exercise using whole‐body periodic acceleration enhances blood supply to ischemic hindlimb. Arteriosclerosis, Thrombosis, and Vascular Biology, 31, 2872–2880. 21940947 10.1161/ATVBAHA.111.229773

[phy215418-bib-0052] Sackner, M. , & Adams, J. A. (2017). Does fibromyalgia sit in a chair? Symptomatic relief with a simulated jogging. Fibromyalgia: Open Access, 2, 1–5.

[phy215418-bib-0053] Sackner, M. A. , Gummels, E. , & Adams, J. A. (2005a). Nitric oxide is released into circulation with whole‐body, periodic acceleration. Chest, 127, 30–39. 15653959 10.1378/chest.127.1.30

[phy215418-bib-0054] Sackner, M. A. , Gummels, E. , & Adams, J. A. (2005b). Effect of moderate‐intensity exercise, whole‐body periodic acceleration, and passive cycling on nitric oxide release into circulation. Chest, 128, 2794–2803. 16236957 10.1378/chest.128.4.2794

[phy215418-bib-0055] Sackner, M. A. , Patel, S. , & Adams, J. A. (2019). Changes of blood pressure following initiation of physical inactivity and after external addition of pulses to circulation. European Journal of Applied Physiology, 119, 201–211. 30350153 10.1007/s00421-018-4016-7 PMC6342894

[phy215418-bib-0056] Sansbury, B. E. , Cummins, T. D. , Tang, Y. , Hellmann, J. , Holden, C. R. , Harbeson, M. A. , Chen, Y. , Patel, R. P. , Spite, M. , Bhatnagar, A. , & Hill, B. G. (2012). Overexpression of endothelial nitric oxide synthase prevents diet‐induced obesity and regulates adipocyte phenotype. Circulation Research, 111, 1176–1189. 22896587 10.1161/CIRCRESAHA.112.266395 PMC3707504

[phy215418-bib-0057] Schuler, G. , Adams, V. , & Goto, Y. (2013). Role of exercise in the prevention of cardiovascular disease: results, mechanisms, and new perspectives. European Heart Journal, 34, 1790–1799. 23569199 10.1093/eurheartj/eht111

[phy215418-bib-0058] Stergiou, G. S. , Palatini, P. , Parati, G. , O'Brien, E. , Januszewicz, A. , Lurbe, E. , Persu, A. , Mancia, G. , Kreutz, R. , & European Society of Hypertension Council and the European Society of Hypertension Working Group on Blood Pressure Monitoring and Cardiovascular Variability . (2021). 2021 European Society of Hypertension practice guidelines for office and out‐of‐office blood pressure measurement. Journal of Hypertension, 39, 1293–1302.33710173

[phy215418-bib-0059] Tenopoulou, M. , & Doulias, P. T. (2020). Endothelial nitric oxide synthase‐derived nitric oxide in the regulation of metabolism. F1000Research, 9, 1–10. 10.12688/f1000research.19998.1 PMC7531049 33042519

[phy215418-bib-0060] Tran, N. , Garcia, T. , Aniqa, M. , Ali, S. , Ally, A. , & Nauli, S. M. (2022). Endothelial nitric oxide synthase (eNOS) and the cardiovascular system: In physiology and in disease states. American Journal of Biomedical Science and Research, 15, 153–177. 35072089 PMC8774925

[phy215418-bib-0061] Trinity, J. D. , Groot, H. J. , Layec, G. , Rossman, M. J. , Ives, S. J. , Morgan, D. E. , Gmelch, B. S. , Bledsoe, A. , & Richardson, R. S. (2015). Passive leg movement and nitric oxide‐mediated vascular function: The impact of age. American Journal of Physiology, 308, H672–H679. 25576629 10.1152/ajpheart.00806.2014 PMC4360052

[phy215418-bib-0062] Trinity, J. D. , Groot, H. J. , Layec, G. , Rossman, M. J. , Ives, S. J. , Runnels, S. , Gmelch, B. , Bledsoe, A. , & Richardson, R. S. (2012). Nitric oxide and passive limb movement: A new approach to assess vascular function. The Journal of Physiology, 590, 1413–1425. 22310310 10.1113/jphysiol.2011.224741 PMC3382331

[phy215418-bib-0063] Trinity, J. D. , Kwon, O. S. , Broxterman, R. M. , Gifford, J. R. , Kithas, A. C. , Hydren, J. R. , Jarrett, C. L. , Shields, K. L. , Bisconti, A. V. , Park, S. H. , Craig, J. C. , Nelson, A. D. , Morgan, D. E. , Jessop, J. E. , Bledsoe, A. D. , & Richardson, R. S. (2021). The role of the endothelium in the hyperemic response to passive leg movement: Looking beyond nitric oxide. American Journal of Physiology, 320, H668–H678. 33306447 10.1152/ajpheart.00784.2020 PMC8082797

[phy215418-bib-0064] Uryash, A. , Bassuk, J. , Kurlansky, P. , Altamirano, F. , Lopez, J. R. , & Adams, J. A. (2015). Antioxidant properties of whole body periodic acceleration (pGz). PLoS One, 10, e0131392. 26133377 10.1371/journal.pone.0131392 PMC4489838

[phy215418-bib-0065] Uryash, A. , Wu, H. , Bassuk, J. , Kurlansky, P. , Sackner, M. A. , & Adams, J. A. (2009). Low‐amplitude pulses to the circulation through periodic acceleration induces endothelial‐dependent vasodilatation. Journal of Applied Physiology (1985), 106, 1840–1847. 10.1152/japplphysiol.91612.2008 19325024

[phy215418-bib-0066] Vanhoutte, P. M. (2018). Nitric oxide: From good to bad. Annals of Vascular Diseases, 11, 41–51. 29682106 10.3400/avd.ra.17-00134 PMC5882356

[phy215418-bib-0067] Wang, Y. X. , Liu, H. B. , Li, P. S. , Yuan, W. X. , Liu, B. , Liu, S. T. , & Qin, K. R. (2019). ROS and NO dynamics in endothelial cells exposed to exercise‐induced wall shear stress. Cellular and Molecular Bioengineering, 12, 107–120. 31719902 10.1007/s12195-018-00557-w PMC6816775

[phy215418-bib-0068] Weinberg, P. D. , Habens, F. , Kengatharan, M. , Barnes, S. E. , & Matz, J. (2001). Characteristics of the pulse waveform during altered nitric oxides ynthesis in the rabbit. British Journal of Pharmacology, 133, 361–370. 11375252 10.1038/sj.bjp.0704084 PMC1572794

[phy215418-bib-0069] Weinberg, P. D. , Habens, F. , Kengatharan, M. , Barnes, S. E. , Matz, J. , Anggard, E. E. , & Carrier, M. J. (2001). Characteristics of the pulse waveform during altered nitric oxide synthesis in the rabbit. British Journal of Pharmacology, 133, 361–370. 11375252 10.1038/sj.bjp.0704084 PMC1572794

[phy215418-bib-0070] Wu, H. , Uryash, A. , Bassuk, J. , Kurlansky, P. , Giridharan, G. A. , Shakeri, M. , Estrada, R. , Sethu, P. , & Adams, J. A. (2012). Mechanisms of periodic acceleration induced endothelial nitric oxide synthase (eNOS) expression and upregulation using an in vitro human aortic endothelial cell model. Cardiovascular Engineering and Technology, 3, 292–301.

[phy215418-bib-0071] Zhang, Q. J. , McMillin, S. L. , Tanner, J. M. , Palionyte, M. , Abel, E. D. , & Symons, J. D. (2009). Endothelial nitric oxide synthase phosphorylation in treadmill‐running mice: Role of vascular signalling kinases. The Journal of Physiology, 587, 3911–3920. 19505983 10.1113/jphysiol.2009.172916 PMC2746618

[phy215418-bib-0072] Zhang, X. , & Gao, F. (2021). Exercise improves vascular health: Role of mitochondria. Free Radical Biology and Medicine, 177, 347–359. 34748911 10.1016/j.freeradbiomed.2021.11.002

