# Supplementary material for: The effects of a motorized passive simulated jogging device on descent of the arterial pulse waveform dicrotic notch: A single arm placebo‐controlled cross‐over trial
Source: Physiol Rep. 2022 Aug 3;10(15):e15418. doi: 10.14814/phy2.15418 (PMC9350470; doi:10.14814/phy2.15418)
Supplement: Supplementary file 1 — Data S1 [file PHY2-10-e15418-s001.pdf]

Electronic Supplemental Material File (ESM)

The Effects of a Motorized Passive Simulated Jogging Device on Descent of the Arterial Pulse Waveform Dicrotic Notch; A Single Arm Placebo-Controlled Cross-Over Trial

Jose A Adams[1*] Jose R Lopez[2] Vinay Nadkarni [3], Zarazuela Zolkipli-Cunningham[4], Harry Ischiropoulos[5] Marvin A Sackner[2] †

1 Division Neonatology Mt Sinai Medical Center of Greater Miami, Miami Beach, Fla, 33140 USA.

2. Mount Sinai Medical Center of Greater Miami, Miami Beach, Fla, 33140 USA.

3. Critical Care, and Pediatrics, The Children's Hospital of Philadelphia, University of Pennsylvania Perelman School of Medicine, Philadelphia, PA 19104, USA.

4 Mitochondrial Medicine Frontier Program (MMFP), Center for Mitochondrial and Epigenomic Medicine (CMEM), The Children's Hospital of Philadelphia, University of Pennsylvania Perelman School of Medicine, Philadelphia, PA 19104, USA.

5. Children's Hospital of Philadelphia Research Institute and Division of Neonatology, Departments of Pediatrics and Systems Pharmacology and Translational Therapeutics, the Raymond and Ruth Perelman School of Medicine, University of Pennsylvania, Philadelphia, PA 19104, USA.

*Corresponding author

E-mail: Tony@msmc.com

Short Title: JD and Pulse Waveform

July 6 ,2022

Fig 1 S

Study A: ***Acute Effects of JD on Blood Pressure and DN position***

A Single Arm Placebo Controlled Cross-Over Trial

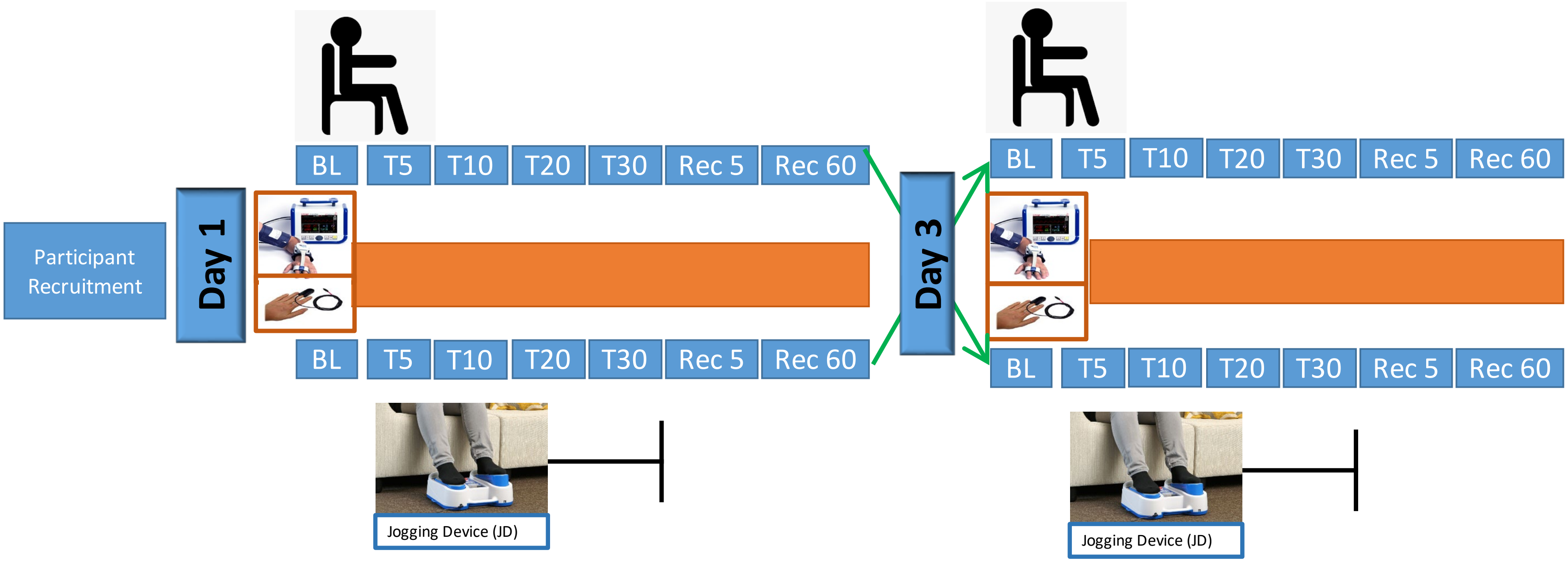

Legend: Recruitment of study subjects was done from a larger protocol ClinicalTrials.gov (NCT03426774). All subjects were studied in the seated posture. On Day 1 subjects were randomized to JD or Control (CONT). On Day 3 subjects were crossed over to receive JD if they started day 1 in CONT, or to CONT if they started day1 on JD. Continuous non-invasive arterial blood pressure (CNAP) and pulse waveform from finger plethysmograph were continuously collected. Measurements of arterial blood pressure and descent of the dicrotic notch (a/b) on the pulse waveform were analyzed after 10 min of quiet sitting (Baseline, BL). The Jogging Device (JD) was begun at a frequency of 190 steps in place. Blood pressure and pulse waveform were analyzed after 5,10,20,30 min of the JD or CONT (T5,T10,T20,T30), and after 5 and 60 min after discontinuation of the JD or CONT (Recovery, REC 5, REC 60).

Consort Flow Diagram Study A

Fig 2 S

Study A: ***Acute Effects of JD on Blood Pressure and DN position***

A Single Arm Placebo Controlled Cross-Over Trial

CONSORT FLOW DIAGRAM

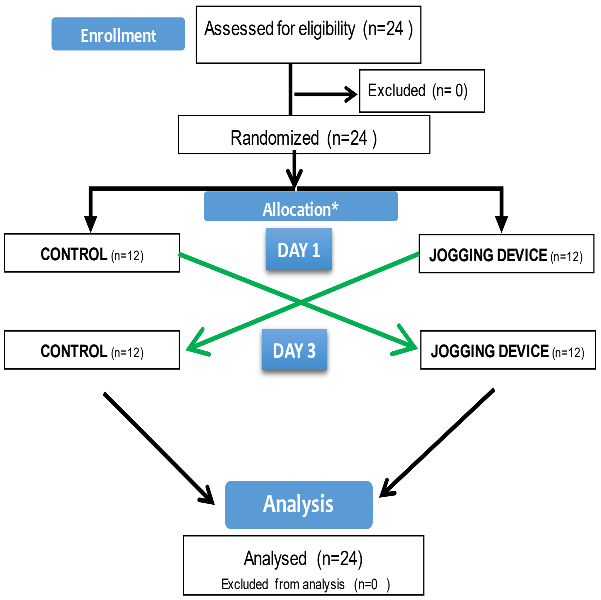

**Georgiev G.Z., "Random Choice Generator", [online] Available at: https://www.gigacalculator.com/randomizers/random-choice-generator.php*

Legend: Twenty-four subjects were enrolled and randomized (using random choice generator*) on day 1 to CONT or JD and crossed over on day 3. No subjects were excluded and all 24 subjects analyzed.

Fig 3 S

Study B: ***Effects of 7 day use of JD on Blood Pressure and DN position***
An Observational Trial

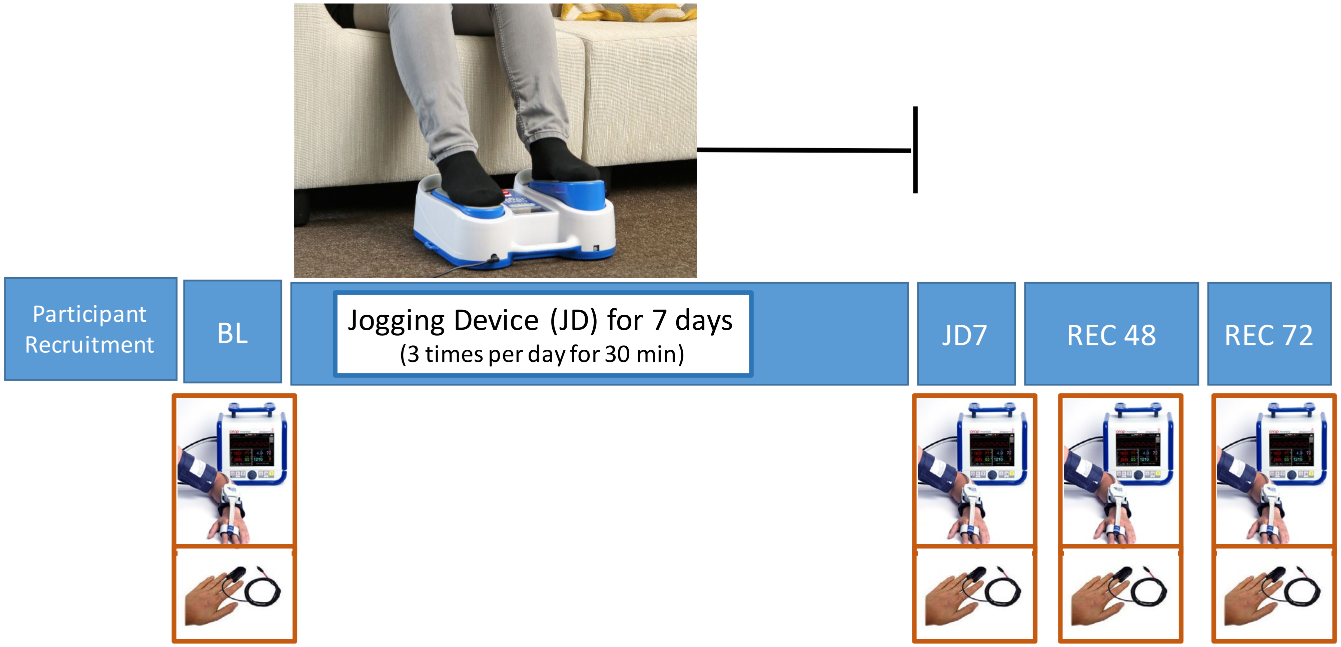

Legend: Recruitment of study subjects was performed from a larger ClinicalTrials.gov ClinicalTrials.gov protocol (NCT03550105). All subjects were studied in seated posture. On day 1, after 10 min of quiet sitting baseline (BL), continuous noninvasive arterial blood pressure (CNAP) and pulse waveform from finger plethysmograph were continuously collected. The subjects were then instructed on the use of the Jogging Device (JD) and instructed to use it at least three times per day for 30 minutes for 7 days (JD7). CNAP and the pulse waveform of the finger plethysmograph were again measured on JD7 and 48 and 72 hours after discontinuation of JD. To replicate real-world behavior, subjects were told to continue their current schedule, diet, and physical activity.

Consort Flow Diagram Study B

Fig 4 S

Study B: ***Effects of 7 day use of JD on Blood Pressure and DN position***

An Observational Trial

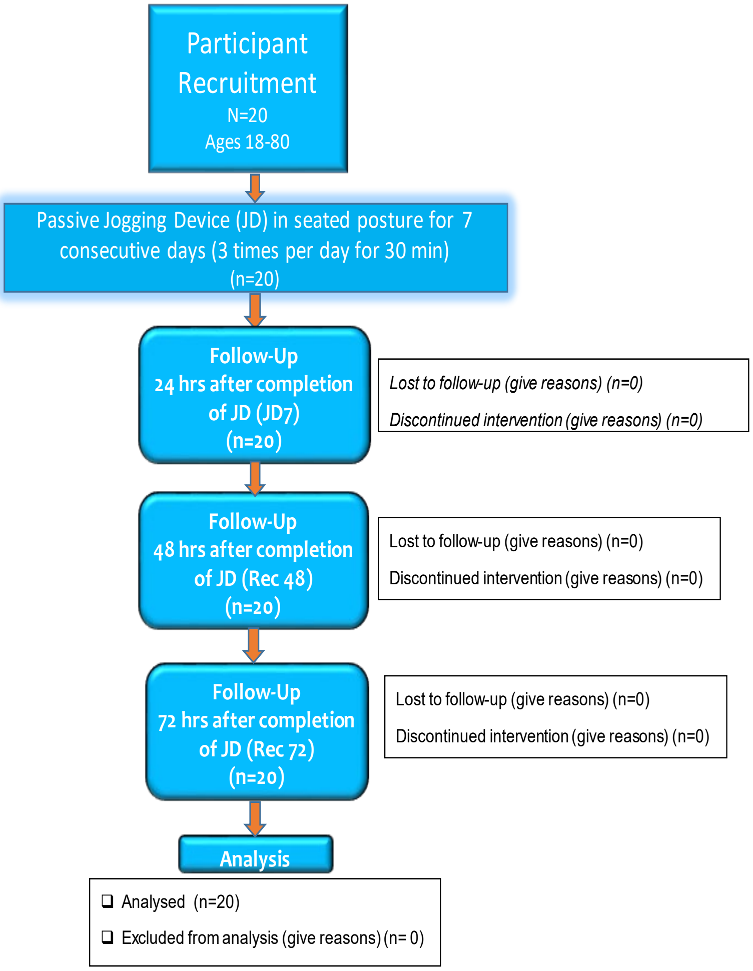

Legend: Twenty subjects were recruited as part of a larger ClinicalTrials.gov protocol (NCT03550105), ages 18-80. Subjects were instructed on the use of the Jogging Device (JD) and instructed to use it at least three times per day for 30 min each session for 7 days (JD7). Three follow-up visits were made in which continuous noninvasive arterial pressure and plethysmographic pulse waveform were measured. There were no excluded subjects and none were lost to follow-up. All subjects were included in the analysis.

JD and Pulse Waveform

CONSORT 2010 checklist of information to include when reporting a randomised trial*

Section/Topic	Item No	Checklist item	Reported on page No
**Title and abstract**			
	1a	Identification as a randomised trial in the title	1
	1b	Structured summary of trial design, methods, results, and conclusions (for specific guidance see CONSORT for abstracts)	2-3
**Introduction**			
Background and objectives	2a	Scientific background and explanation of rationale	5
	2b	Specific objectives or hypotheses	6
**Methods**			
Trial design	3a	Description of trial design (such as parallel, factorial) including allocation ratio	6-8 and ESM
	3b	Important changes to methods after trial commencement (such as eligibility criteria), with reasons	N/A
Participants	4a	Eligibility criteria for participants	7
	4b	Settings and locations where the data were collected	7-8
Interventions	5	The interventions for each group with sufficient details to allow replication, including how and when they were actually administered	8 and ESM
Outcomes	6a	Completely defined pre-specified primary and secondary outcome measures, including how and when they were assessed	8
	6b	Any changes to trial outcomes after the trial commenced, with reasons	N/A
Sample size	7a	How sample size was determined	9
	7b	When applicable, explanation of any interim analyses and stopping guidelines	N/A
Randomisation:			
Sequence generation	8a	Method used to generate the random allocation sequence	N/A
	8b	Type of randomisation; details of any restriction (such as blocking and block size)	N/A
Allocation concealment mechanism	9	Mechanism used to implement the random allocation sequence (such as sequentially numbered containers), describing any steps taken to conceal the sequence until interventions were assigned	N/A
Implementation	10	Who generated the random allocation sequence, who enrolled participants, and who assigned participants to interventions	N/A

Blinding	11a	If done, who was blinded after assignment to interventions (for example, participants, care providers, those assessing outcomes)	N/A
	11b	If relevant, description of the similarity of interventions	N/A
Statistical methods	12a	Statistical methods used to compare groups for primary and secondary outcomes	9
	12b	Methods for additional analyses, such as subgroup analyses and adjusted analyses	9
**Results**			
Participant flow (a diagram is strongly recommended)	13a	For each group, the numbers of participants who were randomly assigned, received intended treatment, and were analysed for the primary outcome	ESM
	13b	For each group, losses and exclusions after randomisation, together with reasons	ESM
Recruitment	14a	Dates defining the periods of recruitment and follow-up	7
	14b	Why the trial ended or was stopped	N/A
Baseline data	15	A table showing baseline demographic and clinical characteristics for each group	25-27
Numbers analysed	16	For each group, number of participants (denominator) included in each analysis and whether the analysis was by original assigned groups	25-27
Outcomes and estimation	17a	For each primary and secondary outcome, results for each group, and the estimated effect size and its precision (such as 95% confidence interval)	10
	17b	For binary outcomes, presentation of both absolute and relative effect sizes is recommended	N/A
Ancillary analyses	18	Results of any other analyses performed, including subgroup analyses and adjusted analyses, distinguishing pre-specified from exploratory	10
Harms	19	All important harms or unintended effects in each group (for specific guidance see CONSORT for harms)	N/A
**Discussion**			
Limitations	20	Trial limitations, addressing sources of potential bias, imprecision, and, if relevant, multiplicity of analyses	15
Generalisability	21	Generalisability (external validity, applicability) of the trial findings	15
Interpretation	22	Interpretation consistent with results, balancing benefits and harms, and considering other relevant evidence	15
**Other information**			
Registration	23	Registration number and name of trial registry	4
Protocol	24	Where the full trial protocol can be accessed, if available	4 and 17
Funding	25	Sources of funding and other support (such as supply of drugs), role of funders	18

Table 1 S

Number and Percent of Subjects by Category of Percent Change from Baseline of the Dicrotic Notch Position (a/b)

**Percent Change in a/b from Baseline**				
	**< 49%**	**50-100%**	**>100%**	**Totals**
Study A (n=24)	5(21%)	6(25%)	13(54%)	24(100%)
Study B (n=20)	3(15%)	11(55%)	6(30%)	20(100%)

Legend:

Categories for the percent change from the baseline of the dicrotic notch (a/b) on the pulse waveform for Study A (Acute Effects of JD on Blood Pressure and DN position) and Study B ((Effects of 7-day use of JD on Blood Pressure and DN position baseline). The percent change for Study A was calculated from the difference between BL and after 30 min of JD (Jogging device, T30). The percent change for Study B was calculated from the difference between BL and 7 days of JD (JD7). The categories are less than 49% change (< 49%) to more than 100% (> 100%) change. In each category, the actual number of study subjects followed by the percent (actual number/study n). In study A, 79% of the study participants had a greater than 50% change in a/b. In Study B, 85% of the study participants had a greater than 50% change in a/b.

FIGURE 5 S

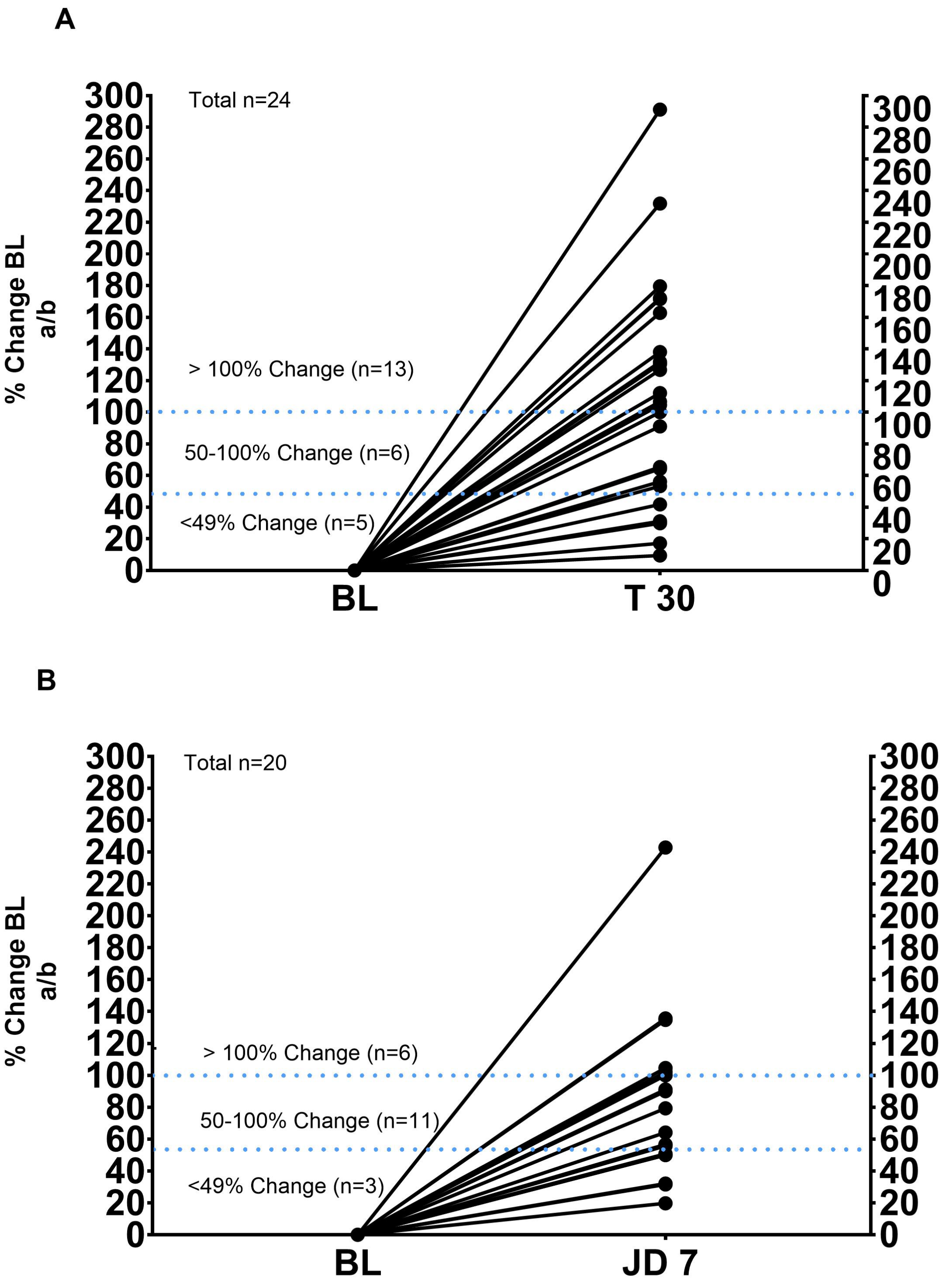

Legend: Panel A: Percent change from baseline (BL) in a/b for Study A (BL to 30 min of JD). Panel B: Percent change from baseline (BL) in a/b for study B (BL to 7 days of JD (JD7). Total number of participants (Total n). The dotted blue lines represent the limit for each category change (e.i more than 100% change, 50-100% change and less than < 49% change) with the respective number of subjects in each category (n=)

Table 2S ***Participant's Characteristics Study A;*** *Acute Effects of JD on Blood Pressure and DN position. Percentage Change from Baseline in a/b in response to JD*

			% Change from BL in a/b Response to JD		
**Subject No**	**Gender**	**Age Range (yrs)**	**<49%**	**50-100%**	**>100%**
1	M	50-55	X		
2	F	45-50			X
3	F	47-52		X	
4	F	36-41			X
5	F	47-52			X
6	M	26-31		X	
7	F	40-45			X
8	F	26-31		X	
9	F	33-38			X
10	F	39-44			
11	F	42-47			X
12	M	23-28		X	
13	F	42-47			X
14	M	55-60			X
15	M	63-68		X	
16	F	85-88	X		
17	M	56-61			X
18	F	56-61			X
19	F	59-64		X	
20	F	63-68	X		
21	F	64-69	X		
22	M	63-68	X		
23	M	58-63			X

24	M	55-60			**X**
			5	6	13

Legend: Participant's Characteristics of the participant of the participant for Study A ; Gender (M=male, F=Female), Age Range in years, and percent change in the a / b category in response to JD (less than 49% change < 49%, 50-100% change and > 100% change). Note 79% (19/24) subjects had a greater than 50% change in a/b from baseline in response to JD.

Table 3S Participant's Characteristics Study B; *Effects of 7 day use of JD on Blood Pressure and DN position Percentage Change from Baseline in a/b in response to JD*

			% Change from BL in a/b Response to JD		
**Subject No**	**Gender**	**Age Range (yrs)**	**<49%**	**50-100%**	**>100%**
1	F	35-45		**X**	
2	F	35-45	**X**		
3	M	60-70	**X**		
4	F	75-85		**X**	
5	M	60-70		**X**	
6	F	60-70		**X**	
7	M	50-60		**X**	
8	F	60-70			**X**
9	F	60-70	**X**		
10	M	55-65			**X**
11	M	60-65		**X**	
12	M	30-35		**X**	
13	F	50-55		**X**	
14	F	30-35		**X**	
15	F	25-30			**X**
16	M	30-35		**X**	
17	F	25-30		**X**	
18	M	60-65			**X**
19	F	40-45			**X**
20	F	45-50			**X**
			**3**	**11**	**6**

Legend: Participant's Characteristic of the participant of the participant for study B; Gender (M = male, F = female), Age range in years,and percent change in the a / b category in response to JD (less than 49% change < 49%, 50-100% change and > 100% change). Note 85% (17/20) subjects had a greater than 50% change in a/b from baseline in response to JD.

Table 4 S

Effect Size of the Jogging Device (JD) Computed Using Cohen's d

**Cohen's d**						
**Study A**	***T5***	***T10***	***T20***	***T30***	***REC 5***	***REC 60***
BPs	0.83	0.83	0.90	1.05	1.08	1.08
BPd	0.67	0.83	0.83	0.94	1.08	0.97
a/b	1.17	1.17	1.18	1.28	1.23	1.30
**Study B**	***JD7***	***REC 48***	***REC 72***			
BPs	1.0	0.96	0.96			
BPd	0.66	0.59	0.68			
a/b	1.63	1.44	1.24			

Legend:

Effect size of JD computed using Cohen's d for each study time. Systolic and diastolic blood pressure (BPs, BPd) and dicrotic notch position a/b. Study A, after 5,10,20, 30 min after JD use (T5,10,20,30) and after 5 and 60 min after discontinuation of JD (REC 5, REC60). Study B, after 7 days of JD use (JD7) and 48 and 72 h after discontinuation of 7 days of JD (REC48, REC72). Cohen's d = 0.2 is considered a small effect size, 0.5 represents a medium effect size, and 0.8 and greater a large effect size. A large effect size of JD (d >1.0) was obtained for a/b for all time periods in both studies.
